# Vulvar Squamous Cell Carcinoma: A Retrospective Analysis of Epidemiologic Characteristics, HPV Status, and Surgical Outcomes in 35 Cases

**DOI:** 10.3390/life15111781

**Published:** 2025-11-20

**Authors:** Daniela Marinescu, Laurențiu Augustus Barbu, Tiberiu Stefăniță Țenea Cojan, Ștefania Tudorache, Dominic Iliescu, Răzvan Alexandru Marinescu, Lucian George Zorilă, Valeriu Șurlin

**Affiliations:** 1 Department of Surgery, Emergency County Hospital, University of Medicine and Pharmacy of Craiova, 2 Petru Rares Street, 200349 Craiova, Romania; dtmarinescu@yahoo.com (D.M.); vsurlin@gmail.com (V.Ș.); 2 Department of Surgery, Railway Clinical Hospital Craiova, University of Medicine and Pharmacy of Craiova, 2 Petru Rares Street, 200349 Craiova, Romania; tiberiu.tenea@umfcv.ro; 3 Department of Obstetrics and Gynecology, University of Medicine and Pharmacy of Craiova, 200349 Craiova, Romania; stefania.tudorache@umfcv.ro (Ș.T.); dominic.iliescu@umfcv.ro (D.I.); zorilalucian@gmail.com (L.G.Z.); 4 Department of Plastic Surgery, University of Medicine and Pharmacy of Craiova, 200349 Craiova, Romania; razvanalexandrumarinescu98@gmail.com

**Keywords:** vulvar squamous cell carcinoma, HPV, epidemiology, surgery, complications, Eastern Europe, de-escalation, morbidity, quality of life

## Abstract

**Background:** Vulvar squamous cell carcinoma (VSCC) is an uncommon yet increasingly relevant malignancy characterized by two distinct etiopathogenetic pathways: HPV-associated and HPV-independent. Data from Eastern Europe remain scarce, where demographic and diagnostic variability may influence disease presentation and outcomes. **Purpose:** This study aimed to assess the epidemiologic characteristics, HPV status, surgical management, and postoperative morbidity of VSCC in a Romanian single-center cohort, providing real-world evidence from an underrepresented region. **Methods:** A retrospective analysis was conducted on all 35 consecutive patients with histologically confirmed vulvar squamous cell carcinoma (VSCC) diagnosed and treated between January 2017 and December 2024 at the Department of Obstetrics and Gynecology, County Emergency Clinical Hospital of Craiova, Romania. Demographic, histopathologic, and surgical data were reviewed. HPV genotyping was performed on formalin-fixed paraffin-embedded (FFPE) tissue using PCR-based methods. **Results:** HPV DNA was detected in 31.4% of cases, predominantly genotypes 16, 18, and 33. HPV-positive patients were significantly younger than HPV-negative ones (median 58 vs. 72.5 years, *p* < 0.001), supporting the dual-pathway model of carcinogenesis. Early postoperative complications occurred in 65.7% of patients and late morbidity in 71.4%, secondary lymphedema. Surgical radicality was not significantly associated with early complications or length of hospitalization. **Conclusions:** This study highlights the epidemiologic and surgical patterns of VSCC in an Eastern European population, showing that conservative surgical strategies can maintain oncologic safety while reducing morbidity. These findings emphasize the need for standardized HPV testing, optimized perioperative care, and improved surveillance programs to enhance outcomes and survivorship.

## 1. Introduction

Vulvar cancer is a rare gynecological malignancy, accounting for ~5% of all female genital tract cancers, but its incidence has been increasing in recent decades, particularly among younger women [1,2,3]. Epidemiological studies from the United States and Europe report a rise in vulvar intraepithelial neoplasia and invasive vulvar squamous cell carcinoma (VSCC), with significant contributions from persistent infection with high-risk human papillomavirus (HPV) [4,5,6]. Globally, HPV 16 has been detected in nearly half of vulvar SCCs, while HPV 18 plays a minor role [4,7]. Two distinct etiopathogenetic pathways are recognized: an HPV-related form, frequently basaloid or warty carcinomas in younger women, and an HPV-independent form, often keratinizing carcinomas associated with chronic dermatoses such as lichen sclerosus [8,9]. This dual pathway concept has significant implications for prevention and treatment, as HPV vaccination has the potential to reduce HPV-driven cases [8,10].

Although VSCC predominantly affects older, postmenopausal women, recent data show a disproportionate increase among younger patients. North American and European registry analyses have documented a 30–40% rise in women under 60 years, whereas incidence rates in older cohorts remained stable [11,12]. Beyond HPV, additional risk factors include cigarette smoking, immunosuppression (e.g., HIV infection), and chronic inflammatory dermatoses, all of which contribute to disease development and progression [13].

No formal screening program exists for vulvar cancer, and early diagnosis relies on prompt biopsy of suspicious lesions [13]. The recognition of precursors has also evolved, with current terminology distinguishing between HPV-related high-grade squamous intraepithelial lesions (HSIL) and HPV-independent differentiated vulvar intraepithelial neoplasia (dVIN). HSIL typically progresses slowly and is strongly linked to HPV 16, while dVIN carries a higher malignant potential and more rapid progression to invasive disease [13,14].

The role of prophylactic HPV vaccination is particularly important, as randomized trials and population-based studies demonstrate >90% efficacy in preventing HPV-related vulvar intraepithelial neoplasia [13,15]. These findings suggest that widespread vaccination programs may reduce not only cervical cancer but also the incidence of HPV-driven VSCC.

Traditionally, radical vulvectomy with bilateral inguinofemoral lymphadenectomy was the standard treatment, but it is associated with considerable morbidity, including lymphedema, wound breakdown, chronic pain, and impaired sexual function [4,11]. In recent years, less radical approaches such as hemivulvectomy, sentinel lymph node biopsy, and minimally invasive lymphadenectomy have been introduced to balance oncological safety with quality of life [8,13]. However, most existing data on VSCC derive from Western Europe and North America, while information from Eastern European populations remains scarce. This gap in regional evidence highlights the need for updated data on epidemiologic patterns, HPV-related mechanisms, and treatment outcomes in VSCC. Molecular characterization, HPV distribution, and morbidity outcomes are inconsistently reported in regional series. This study was therefore designed to address these gaps and to contextualize current management within a regional clinical framework.

This study aims to describe the epidemiologic characteristics and HPV-related molecular mechanisms, and to synthesize current advances in management approaches for vulvar squamous cell carcinoma. Taken together, these perspectives provide an integrated understanding of contemporary clinical patterns and therapeutic implications in VSCC. Despite being the fourth most common gynecologic malignancy, VSCC remains underrepresented in international cancer research, with relatively few high-quality studies and limited randomized data. This scarcity of evidence underscores the need for continuous reporting and collaborative efforts to refine treatment algorithms. By integrating current epidemiologic data with surgical and adjuvant management perspectives, our work aims to highlight knowledge gaps and emphasize the importance of ongoing research in improving outcomes for women with this uncommon but clinically significant disease. Specifically, the study aimed to describe the epidemiologic characteristics and distribution patterns (age at diagnosis, disease stage, and temporal distribution), HPV status (HPV-related vs. HPV-independent cases), surgical management approaches (extent of resection and lymph node assessment), and postoperative outcomes (complications and recurrence rates) in a real-world Eastern European population.

## 2. Materials and Methods

### 2.1. Study Design and Setting

This retrospective observational cohort study included 35 consecutive patients diagnosed with vulvar squamous cell carcinoma (VSCC) and treated between January 2017 and December 2024 at the Department of Obstetrics and Gynecology, County Emergency Clinical Hospital of Craiova, Romania.

### 2.2. Inclusion and Exclusion Criteria

Patients were eligible for inclusion if they had a histologically confirmed primary vulvar squamous cell carcinoma (VSCC) of FIGO stage I–IV, were aged 18 years or older, had complete clinicopathologic and surgical data, and had available formalin-fixed paraffin-embedded (FFPE) tissue blocks suitable for HPV testing (Table 1). Exclusion criteria comprised non-squamous histologic subtypes (including melanoma, sarcoma, Paget disease, or adnexal tumors), recurrent or metastatic disease, prior treatment at another institution, and incomplete or missing clinical documentation. All cases were diagnosed and managed at the same institution during the study period.

**Figure 1:**
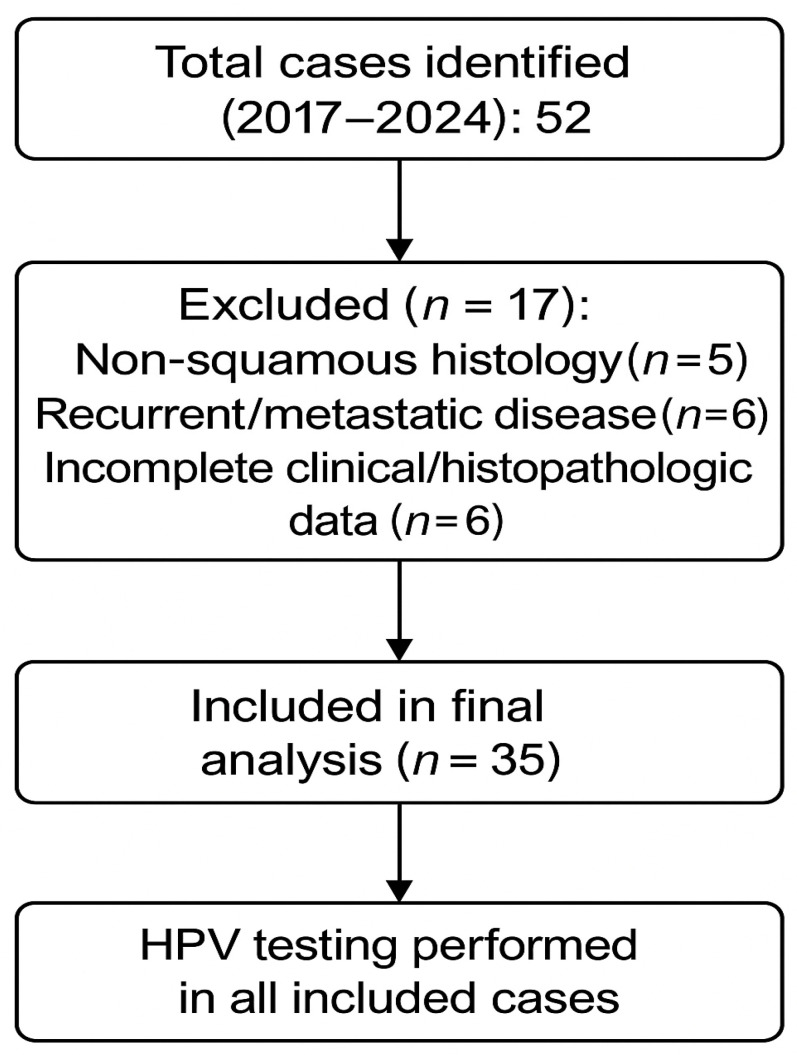
PRISMA flow diagram of patient selection.

A total of 52 cases were identified between 2017 and 2024. Seventeen cases were excluded (non-squamous histology, recurrent/metastatic disease, or incomplete data), and 35 were included in the final analysis (Figure 1). HPV testing was performed in all included cases.

Baseline clinicopathologic characteristics of the study cohort are summarized in Table 2.

Epidemiologic characteristics were described according to patient age, tumor stage, and year of diagnosis. HPV status was determined by real-time PCR genotyping using the Anyplex™ II HPV28 Detection Kit on FFPE tissue samples. Surgical management was categorized by type of resection and extent of lymph node dissection. Postoperative outcomes included complication rates and recurrence.

### 2.3. Clinicopathologic and Histopathologic Evaluation

Clinical variables recorded included age, tumor site, lesion size, histologic subtype, grading, lymphovascular invasion, resection margins, and lymph node involvement. Tumors were staged according to the 2021 International Federation of Gynecology and Obstetrics (FIGO) classification. Histopathological evaluation was performed on hematoxylin–eosin–stained slides by two independent pathologists, according to the 2020 WHO classification of vulvar tumors. Tumor grading (G1–G3) followed the WHO criteria, where G1 indicates well-differentiated, G2 moderately differentiated, and G3 poorly differentiated squamous cell carcinoma. The presence of associated precursor lesions or chronic inflammatory dermatoses (lichen sclerosus, differentiated VIN) was also documented when available.

### 2.4. HPV Detection and Genotyping

HPV DNA testing was performed on archived formalin-fixed, paraffin-embedded (FFPE) tissue blocks. DNA extraction was carried out using the QIAamp DNA FFPE Tissue Kit (Qiagen, Hilden, Germany). HPV detection and genotyping were performed using real-time PCR with the Anyplex™ II HPV28 Detection Kit (Seegene, Seoul, South Korea), which identifies 28 high- and low-risk HPV genotypes. According to the manufacturer’s validation data, the Anyplex™ II HPV28 Detection Kit demonstrates an analytical sensitivity of approximately 10–100 copies per reaction and a clinical sensitivity and specificity exceeding 95% for high-risk HPV types. The multiplex real-time PCR design employs type-specific dual priming oligonucleotide (DPO) technology, which minimizes cross-reactivity among genotypes and ensures reliable discrimination between closely related HPV subtypes. Cases were considered HPV-positive if viral DNA for any high-risk genotype was identified. Immunohistochemical (p16) and RNA-based (E6/E7 mRNA) assays were not available in our institution during the study period, representing a methodological limitation.

### 2.5. Surgical and Postoperative Management

The choice of surgical approach—radical vulvectomy, hemivulvectomy, or separate-incision surgery—was based on tumor size, localization, and margin status, following ESGO–ESMO 2023 recommendations. Sentinel lymph node biopsy (SLNB) was performed for eligible early-stage, unifocal cases. Postoperative data included hospital stay, drainage duration, and complications.

Early (≤30 days) and late (>30 days) postoperative complications were systematically documented using institutional reporting templates and classified according to the Clavien–Dindo system, when applicable. Variables related to comorbidities (diabetes, cardiovascular disease, renal impairment) were collected to assess their association with complication risk. Data consistency was independently verified by two investigators before analysis. All surgical procedures were performed by the same senior gynecologic oncology team, following standardized institutional protocols, minimizing inter-surgeon variability and potential confounding effects on postoperative outcomes.

### 2.6. Statistical Analysis

Variables analyzed included patient age, tumor stage, HPV status, comorbidity profile (diabetes, cardiovascular disease, renal impairment), surgical approach, and postoperative complications. Statistical analyses were performed using IBM SPSS Statistics version 26.0 (IBM Corp., Armonk, NY, USA). Continuous variables were summarized as medians and ranges, and categorical variables as frequencies and percentages. Comparative analyses were conducted using the Mann–Whitney U test for continuous data and the chi-square or Fisher’s exact test for categorical variables. Descriptive and non-parametric statistical tests (Mann–Whitney U, chi-square, and Fisher’s exact tests) were applied to assess associations between clinicopathologic parameters and postoperative complications. Statistical significance was defined as *p* < 0.05. Due to the limited sample size, multivariate regression analysis was not performed to avoid overfitting and unreliable estimates. No formal sample size calculation was performed, as this was a retrospective study that included all eligible patients diagnosed and treated for vulvar squamous cell carcinoma at our institution between 2017 and 2024.

### 2.7. Ethical Approval

The study was conducted in accordance with the Declaration of Helsinki and was approved by the Ethics Committee of the University of Medicine and Pharmacy of Craiova and the County Emergency Clinical Hospital of Craiova (approval no. 47352/3 October 2025). As this was a retrospective observational study based on existing medical records and archived samples, no additional consent was required. All patients had provided written informed consent for diagnostic and therapeutic procedures, including the use of anonymized data for research purposes, at the time of hospital admission.

## 3. Results

Baseline characteristics of the cohort are shown in Table 2. The mean patient age was 68.4 ± 10.0 years (range 43–83). Most tumors were low- or intermediate-grade (G1: 48.6%; G2: 40.0%), and all were squamous cell carcinomas. HPV DNA was detected in 31.4% of cases (11/35), all genotypes 16/18/33; HPV-positive patients were younger (median 58.0 years) than HPV-negative ones (72.5 years). Surgical management included hemivulvectomy in 34.3% of patients, radical total vulvectomy in 17.1%, and radical vulvectomy with separate incisions in 48.6%. Mean hospital stay was 8.8 ± 2.9 days, and drainage duration averaged 11.7 ± 6.6 days. Early postoperative complications included neuropathic pain/paresthesia (31.4%), lymphocysts (17.1%), hematoma (8.6%), wound infection (5.7%) and complete wound dehiscence (2.9%), whereas 34.3% had no early complications. Late morbidity consisted mainly of lymphedema (71.4%), with 2.9% local recurrence; 25.7% had no late complications.

**Figure 2:**
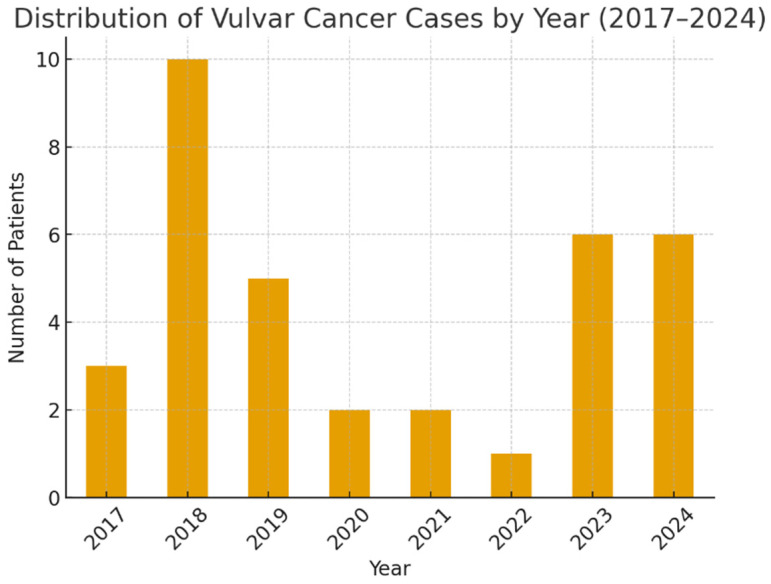
Annual distribution of vulvar cancer cases (2017–2024).

The incidence of vulvar cancer varied between 2017 and 2024, peaking in 2018 (28.6% of cases), declining during 2019–2022, and rising again in 2023–2024 (Figure 2).

Postoperative outcomes are summarized in Table 3. Early complications occurred in 23/35 patients (65.7%), most frequently neuropathic pain/paresthesia (31.4%) and lymphocyst formation (17.1%). Late complications were identified in 28/35 cases (80.0%), predominantly lymphedema (71.4%). Complication rates varied across tumor grade, surgical procedure, age group, and diabetes status, but none of these associations reached statistical significance (early complications: *p* = 0.709; late complications: *p* = 1.000). Local recurrence occurred in one patient (2.9%), corresponding to a G3 tumor.

Postoperative recovery parameters are shown in Table 4. The mean hospital stay was 8.8 ± 2.9 days (median 8; range 5–15), and the mean drainage duration was 11.7 ± 6.6 days (median 10; range 4–29). Cardiovascular comorbidities were present in 32/35 patients and were associated with a mean hospitalization of 8.9 days and a mean drainage duration of 11.9 days. Smoking status and mild chronic kidney disease showed no meaningful differences in recovery metrics. When comparing time periods, patients treated before 2020 had a mean hospital stay of 8.6 days and drainage duration of 10.8 days, whereas the post-2020 cohort had means of 9.0 days and 12.6 days, respectively. Longer drainage duration (≥10 days) was associated with longer hospitalization (10.6 vs. 6.9 days). Recurrence occurred in 1/17 patients with short drainage and in none of the patients with prolonged drainage. Lymphedema was present in 70.6% of patients with short drainage and 72.2% of those with prolonged drainage.

Most tumors had a **vegetative/ulcerated appearance** (91.4%), while three cases were classified as **infiltrative** (Table 5). Infiltrative tumors were predominantly higher grade (G2–G3) and all developed complications, compared with 78.1% of vegetative/ulcerated cases. However, differences in grading distribution (*p* = 1.00) and complication rates (*p* = 1.00) were not statistically significant.

*Statistical tests:*

- Grading distribution across groups: χ^2^ test, *p* = 1.00
- Complication rates across groups: χ^2^ test, *p* = 1.00

Of the 35 patients, 11 (31.4%) were HPV-positive (Table 6). HPV-positive cases were significantly younger than HPV-negative ones (median 58.0 vs. 72.5 years, *p* < 0.001). Tumor grade (*p* = 0.575), early complications (*p* = 0.709), late complications (*p* = 1.000), and hospitalization duration (*p* = 0.474) did not differ significantly between the two groups. After adjustment for age and diabetes, HPV status was not associated with hospital stay length (*p* = 0.804). Active smoking was reported in 4/11 HPV-positive patients (36.4%) and in none of the HPV-negative patients.

## 4. Discussion

### 4.1. Epidemiology and HPV Association

Vulvar squamous cell carcinoma (VSCC) is an uncommon gynecologic malignancy, representing 2–5% of all female genital tract cancers [9,16]. Population-based studies have shown a rising incidence, particularly among younger women, primarily related to human papillomavirus (HPV) exposure [3,17]. Approximately 40–50% of VSCCs are HPV-associated—predominantly HPV16—while the remaining cases follow an HPV-independent pathway often linked to chronic dermatoses such as lichen sclerosus [1,4,9,18,19,20,21,22,23,24,25,26,27,28].

This dual-pathway model distinguishes HPV-related high-grade squamous intraepithelial lesion (HSIL) from HPV-independent differentiated VIN (dVIN), the latter showing higher malignant potential and faster progression to invasive disease [1]. Prophylactic HPV vaccination demonstrates >90% efficacy in preventing HPV-related VIN and is anticipated to reduce the global burden of HPV-driven VSCC [1]. The present study adds regional data to these global observations by characterizing the clinicopathologic and molecular features of VSCC in an Eastern European cohort.

In the present study, HPV DNA was detected in 31.4% of vulvar squamous cell carcinomas (11/35), predominantly genotypes 16, 18, and 33. HPV-positive patients showed a younger age distribution than HPV-negative ones, a pattern consistent with the dual-pathway model of VSCC carcinogenesis. Although this rate is slightly below the 40–50% reported internationally, it may reflect regional demographic or methodological differences. The predominance of HPV-negative tumors in older women reinforces the HPV-independent, keratinizing pathway, often linked to chronic dermatoses such as lichen sclerosus. Similarly to other rare epithelial gynecologic tumors, such as endometrioid adenofibroma of the ovary, accurate subclassification of VSCC depends heavily on integrating morphology with immunohistochemical and molecular findings to delineate distinct pathogenetic mechanisms [29,30].

Tobacco smoking is a recognized cofactor in HPV-related carcinogenesis, promoting viral persistence and epithelial dysplasia through local immunosuppression and oxidative stress. In our cohort, active smoking was identified in 36.4% of HPV-positive patients and was absent among HPV-negative cases, consistent with international data indicating higher smoking prevalence in HPV-driven VSCC [6,31]. Although smoking was not an independent predictor of postoperative morbidity in our cohort, its well-established etiologic role in HPV-related vulvar carcinogenesis highlights the importance of preventive counseling and smoking cessation strategies in this patient population [6,31].

In summary, in our cohort, vulvar squamous cell carcinoma predominantly affected postmenopausal women, with a median age of 68.4 years. HPV DNA was detected in 31.4% of cases, mainly genotypes 16, 18, and 33, confirming the coexistence of HPV-dependent and HPV-independent pathways. HPV-positive tumors occurred in younger patients, whereas HPV-negative, keratinizing forms predominated in older women, consistent with global dual-pathway epidemiologic models.

### 4.2. Surgical Management

#### 4.2.1. Evolution of Surgical Management—Literature Perspective

This subsection provides a brief contextual overview of the evolution of surgical management in vulvar squamous cell carcinoma (VSCC). Historically, radical vulvectomy with bilateral inguinofemoral lymphadenectomy achieved satisfactory oncologic control but caused substantial morbidity, including wound breakdown, chronic lymphedema, and sexual dysfunction [11,30]. As early detection increased, population-based studies demonstrated no survival benefit for en bloc resection compared with less radical surgery when adequate margins and lymph node assessment were achieved [4,7,32]. Consequently, the shift toward individualized, function-preserving surgery marked a major paradigm change in VSCC management. These international trends are consistent with our institutional experience, where conservative resections were preferred whenever oncologically feasible, reflecting the ongoing global transition toward morbidity reduction and quality-of-life preservation.

#### 4.2.2. Separate-Incision Techniques and Hemivulvectomy

Separate-incision vulvectomy and hemivulvectomy emerged as key steps in surgical de-escalation, minimizing groin wound complications while maintaining oncologic safety [11,32]. These techniques preserve vascularity and significantly reduce flap necrosis, dehiscence, and infection compared with classical en bloc procedures. In our series, the predominant surgical approaches were radical vulvar surgery with separate vulvar and inguinal incisions (17.1%) and hemivulvectomy, with or without adjuvant radiotherapy (14–17%). The mean hospital stay was 8.8 days, and mean postoperative drainage duration was 11.7 days. No statistically significant associations were identified between tumor grade (*p* = 0.709) or surgical procedure (*p* = 0.472) and early postoperative morbidity, reinforcing that conservative surgical approaches do not compromise short-term oncologic safety. These findings align with contemporary registry data confirming improved recovery and reduced morbidity with conservative approaches [32,33,34].

#### 4.2.3. Sentinel Lymph Node Biopsy (SLNB)

Sentinel lymph node biopsy (SLNB) has redefined nodal management in early-stage VSCC by reducing morbidity without compromising oncologic safety. The GROINSS-V and GROINSS-V II trials established SLNB as a reliable alternative to complete lymphadenectomy in unifocal tumors <4 cm with clinically negative groins, achieving disease-specific survival comparable to full dissection and markedly fewer complications [1,16]. Our institution adopted selective SLNB based on GROINSS-V criteria, using technetium-99m or ICG fluorescent mapping when appropriate. Consistent with the literature, the approach significantly reduced chronic lymphedema and wound complications while maintaining adequate nodal control, highlighting its essential role in modern conservative management.

#### 4.2.4. Minimally Invasive Inguinofemoral Lymphadenectomy (VEIL/Robotic)

Minimally invasive techniques such as video endoscopic inguinofemoral lymphadenectomy (VEIL) and robot-assisted dissection extend the principles of SLNB by further reducing surgical trauma [35,36]. Although large-scale VSCC-specific data remain limited, early reports demonstrate comparable nodal yields and lower complication rates compared with open surgery. In our practice, the use of separate incisions, preservation of the saphenous vein, and ICG lymphography for lymphatic mapping were progressively implemented since 2020, improving lymphatic control and postoperative outcomes. These refinements align with international recommendations promoting tailored, low-morbidity approaches in appropriately selected patients [1,16].

#### 4.2.5. Morbidity and Strategies for Complication Prevention

Despite advances in technique, morbidity remains a major challenge in VSCC surgery. Historically, up to 40% of patients experienced wound complications or chronic lymphedema after radical procedures [7,36,37]. Modern refinements—including separate vulvar and groin incisions, early mobilization, meticulous wound care, and selective saphenous vein preservation—have substantially improved outcomes [1]. In our cohort, early postoperative complications occurred in 65.7% of patients (mainly neuropathic pain, 31%, and lymphocyst formation, 17%), while late morbidity was noted in 71.4%, dominated by lymphedema. Early complications strongly predicted late sequelae (100% vs. 42%, *p* < 0.05), underscoring the cumulative burden of morbidity and the need for preventive strategies.

#### 4.2.6. Training and Surgical Expertise

Given the anatomical complexity and rarity of VSCC, surgical expertise is often limited, emphasizing the need for centralization in specialized oncologic centers [1,16]. Cadaver-based workshops and structured training programs improve anatomical precision and complication prevention, while registry-based audits enhance adherence to standards [38]. Maintaining proficiency requires at least 5–10 groin dissections annually. Our institutional practice follows this principle, integrating SLNB and lymphatic-sparing approaches within a multidisciplinary framework. Training initiatives focusing on HPV-related versus HPV-independent pathways, as well as survivorship-oriented care, are vital for modern surgical education and outcome optimization [1,16,28].

#### 4.2.7. Vulvoperineal Reconstruction and Quality of Life

Reconstruction is a cornerstone of function-preserving VSCC surgery. Early involvement of reconstructive teams enables tension-free closure, reduces wound breakdown, and restores sexual and urinary function. Commonly used flaps include V–Y advancement, rhomboid, and gluteus maximus myocutaneous flaps [25]. In our institution, flap-based closure was employed in complex resections or after bilateral groin dissection to optimize healing and minimize tension. These strategies align with current ESGO and BGCS recommendations advocating for individualized reconstruction to balance oncologic safety with functional recovery [16].

#### 4.2.8. Advanced and Recurrent Disease

Management of advanced or recurrent VSCC has evolved from radical exenterative procedures toward individualized multimodal therapy. Cross-sectional imaging (MRI/CT, PET-CT) now guides the extent of surgery and adjuvant therapy [16]. For patients unsuitable for resection, chemoradiation offers organ preservation with favorable local control [9]. In our experience, locally advanced tumors were managed through combined surgery and adjuvant radiotherapy, achieving acceptable complication rates and low locoregional recurrence. Centralization, multidisciplinary evaluation, and access to reconstructive and radiation expertise remain essential to balance curative intent with quality-of-life preservation [11,16].

### 4.3. Adjuvant Therapies and Multimodal Treatment

Adjuvant therapy is pivotal in optimizing outcomes for patients with locally advanced or node-positive vulvar squamous cell carcinoma (VSCC). Historically, radical surgery alone yielded suboptimal control, particularly in women with lymph node metastases. Subsequent evidence established that adjuvant radiotherapy significantly improves local control and disease-free survival in patients with nodal involvement or close/positive margins, forming the basis of modern multimodal management that integrates surgery, radiotherapy, and systemic therapy to enhance oncologic outcomes while minimizing morbidity [28,39]. Current guidelines recommend adjuvant radiotherapy—often with concurrent chemotherapy—to the groins and pelvis in cases with pathologically positive inguinofemoral nodes, multiple nodal metastases, or extranodal extension (ECE). Large-scale analyses, including NCDB and AGO-CaRE-1 studies, confirm the greatest benefit when chemotherapy is combined with adjuvant radiation in node-positive disease [1,16]. Typical radiation doses range from ~50 Gy for microscopic metastases to 60–70 Gy for extracapsular or gross residual disease, with IMRT preferred for its superior dose conformity and reduced toxicity [16,35,36]. In our cohort, adjuvant radiotherapy was delivered in high-risk cases, achieving satisfactory locoregional control and acceptable tolerance. The GROINSS-V II trial further demonstrated that, in patients with sentinel node micrometastases, radiotherapy can safely replace completion lymphadenectomy—reducing morbidity without compromising outcomes [16]. Concurrent cisplatin remains the standard radiosensitizer in both adjuvant and definitive settings, while emerging evidence supports potential roles for immunotherapy and HPV-directed therapeutic vaccines in recurrent or metastatic VSCC [10]. Overall, IMRT-based adjuvant radiotherapy and selective chemoradiation have improved the therapeutic index in VSCC, anchoring a personalized multimodal strategy that balances tumor control with quality of life [1,16,28].

### 4.4. Complications and Quality of Life

Postoperative complications remain a major challenge in the management of vulvar squamous cell carcinoma (VSCC). Early complications such as wound infection, dehiscence, lymphocyst formation, and neuropathic pain are particularly frequent after en bloc resections with bilateral inguinofemoral lymphadenectomy, where disruption of lymphatic drainage and flap ischemia delay healing [11,12,40]. Late sequelae—most notably chronic lymphedema (30–40%), persistent neuropathic pain, and psychosexual dysfunction—severely impact quality of life (QoL) [11]. IMRT-based radiotherapy provides superior dose conformity and tissue sparing, improving postoperative tolerance and long-term QoL [1,41].

In our cohort, early postoperative complications occurred in 23/35 patients (65.7%), most commonly neuropathic pain (31%) and lymphocyst formation (17%). Late morbidity was recorded in 28/35 (80.0%), predominantly lower-limb lymphedema. In patients with lymphatic complications, indocyanine green (ICG) fluorescent lymphography enabled targeted repair of ruptured collectors, improving lymphorrhea control and reducing reinterventions. Early complications strongly predicted late sequelae (100% vs. 41.7%, *p* < 0.05). Local recurrence was rare (1/35; 2.9%), limited to one G3 case managed by radical vulvectomy without adjuvant radiotherapy. Greater surgical radicality did not reduce recurrence, supporting guideline-aligned surgical de-escalation and strengthened morbidity-prevention strategies. Older patients (≥65 years) and those with diabetes had higher complication rates (85% vs. 62%) and longer recovery, while cardiovascular comorbidities—present in 91%—were linked to increased morbidity.

These findings reinforce that achieving optimal outcomes in VSCC requires a balance between oncologic safety and functional preservation. Integrated management—including tailored surgery (SLNB), reconstructive planning, advanced radiotherapy, and vigilant care of dVIN or lichen sclerosus—should be centralized in expert centers [1,5,6,15,40]. HPV vaccination and corticosteroid therapy for dVIN have demonstrated preventive potential [5], while early recognition of severe postoperative infections and prompt multidisciplinary management remain vital [42,43]. Collectively, these strategies align with modern survivorship-focused care aiming to reduce cumulative morbidity and improve QoL in women with VSCC.

### 4.5. Future Perspectives

The dual-pathway model of vulvar squamous cell carcinoma (VSCC) emphasizes tailored prevention and management strategies for HPV-associated and HPV-independent disease. For HPV-driven VSCC, primary prevention through vaccination remains the most effective measure. Large-scale population studies demonstrate that HPV vaccination significantly reduces non-cervical premalignant lesions and is expected to lower HPV-related vulvar cancer incidence over time [44]. In HPV-independent disease, differentiated VIN (dVIN) and lichen sclerosus (LS) represent key precursors. Long-term studies confirm that adherence to ultrapotent topical corticosteroid therapy after excision reduces progression risk, supporting structured dermatology–oncology collaboration as an essential component of tertiary prevention [5]. Nevertheless, LS and dVIN remain underrepresented in current research, warranting prospective registries and focused epidemiologic studies [28].

Surgical management continues to evolve toward individualized, function-preserving approaches. Sentinel lymph node biopsy (SLNB) and minimally invasive nodal techniques such as video endoscopic inguinofemoral lymphadenectomy (VEIL) or robotic-assisted dissection achieve oncologic safety with reduced morbidity, particularly in high-volume centers [1,45]. Consolidated incidence trends from the United States (age-standardized rate 2.40/100,000; AAPC +0.8% from 2000 to 2019; highest among women ≥85 years) further justify prevention and surgical de-escalation strategies aimed at preserving quality of life in an aging population [15].

Beyond surgery, systemic innovations are reshaping the therapeutic landscape. Immunotherapy and molecularly targeted agents show promise in advanced and recurrent VSCC, particularly for HPV-related tumors where viral antigens enhance immunogenicity. Emerging HPV-targeted therapeutic vaccines and CRISPR-based genome editing technologies are also under investigation [46]. Future progress depends on longitudinal registries and multicenter collaborations integrating epidemiologic, molecular, and clinical data to refine treatment algorithms and generate robust real-world evidence [28]. In summary, the future of VSCC management relies on comprehensive prevention, precise surgical de-escalation, and the thoughtful integration of emerging systemic therapies to improve survival while preserving function and quality of life.

### 4.6. Strengths and Limitations

The main strength of this study lies in its comprehensive integration of epidemiologic, virologic, and surgical data within a single-center cohort, offering a detailed real-world overview of vulvar squamous cell carcinoma (VSCC) management in an Eastern European population. This regional perspective is particularly valuable, as data from Central and Eastern Europe remain limited, and diagnostic techniques—including HPV detection and molecular stratification—are still evolving. The inclusion of HPV genotyping and its correlation with clinicopathologic and surgical outcomes provides additional insight into the dual-pathway model of carcinogenesis. Furthermore, the systematic documentation of early and late postoperative complications provides evidence-based support for the ongoing shift toward surgical de-escalation and morbidity reduction.

However, several limitations must be acknowledged. The retrospective, single-center design and relatively small sample size—restricted to histologically confirmed squamous cell carcinomas—limit both generalizability and statistical power. Accordingly, the present analysis should be interpreted as a descriptive assessment of epidemiologic and clinical characteristics rather than inferential trends. HPV testing was performed on archived FFPE tissue, and immunohistochemical analyses such as p16 and p53 staining or RNA-based assays (E6/E7 mRNA, RNAscope) were not routinely available. In addition, our findings must be interpreted in the context of regional diagnostic and surveillance constraints. Access to standardized immunohistochemistry and molecular assays remains limited in many Eastern European centers, and there are no dedicated national registries or structured follow-up programs for vulvar cancer. These systemic limitations may contribute to delayed diagnosis, incomplete long-term surveillance, and underreporting of recurrence or survival outcomes, reflecting broader disparities in cancer care infrastructure across the region. This limitation may have reduced the precision of differentiating HPV-dependent (HSIL-like) from HPV-independent (dVIN-like) pathways. Similar challenges have been reported in other epithelial malignancies, such as papillary breast carcinoma, where the absence of standardized immunohistochemical markers can hinder the distinction between in situ and invasive disease, highlighting the decisive role of markers such as SMA, p63, and CK5/6 in diagnostic accuracy [47]. In addition, incomplete patient return for ancillary testing or long-term follow-up further constrained the assessment of survival and recurrence outcomes. Survival analysis was not performed because long-term follow-up data were incomplete for several patients; only one local recurrence was observed during the study period. Rather than identifying novel biological mechanisms, our study provides regional confirmation of globally recognized patterns of carcinogenesis and postoperative morbidity, emphasizing the importance of standardized HPV testing and morbidity-reduction strategies in Eastern European populations.

Despite these limitations, this study contributes valuable real-world evidence from an underrepresented geographic region and underscores the need for standardized HPV testing, consistent immunohistochemical profiling, and long-term surveillance strategies to improve diagnostic precision and survivorship outcomes in VSCC.

This retrospective study reinforces the need for multidisciplinary collaboration and standardized data collection in rare gynecologic malignancies, consistent with our previous regional experience [48]. Applying these principles to vulvar squamous cell carcinoma (VSCC) supports patient-tailored, quality-of-life–oriented care and underscores the importance of developing multicenter registries to harmonize clinical practice in Romania and Eastern Europe.

## 5. Conclusions

This single-center study provides regional insight into the epidemiology, HPV profile, and surgical outcomes of vulvar squamous cell carcinoma (VSCC) in an underrepresented Eastern European population. The findings highlight that conservative, function-preserving surgery can achieve oncologic safety while reducing morbidity. The coexistence of HPV-dependent and HPV-independent pathways underscores the importance of standardized HPV testing and integrated diagnostic strategies. Overall, this work emphasizes the need for centralization of care, multidisciplinary collaboration, and long-term surveillance programs to improve outcomes and quality of life for women with VSCC.

## Figures and Tables

**Table 1:** Inclusion and exclusion criteria for patient selection.

Criterion Type	Details
Inclusion	Primary histologically confirmed VSCC
	FIGO stage I–IV
	Age ≥ 18 years
	Complete clinicopathologic and surgical data
	FFPE tissue blocks available for HPV testing
	Treated at the same institution
Exclusion	Non-squamous histology (melanoma, sarcoma, Paget, adnexal)
	Recurrent/metastatic disease
	Prior treatment elsewhere
	Missing or incomplete documentation

**Table 2:** Baseline clinicopathologic characteristics of patients with vulvar squamous cell carcinoma (VSCC, n = 35).

Variable	n (%) or Value
**Age (years)**	Mean 68.4 ± 10.0 (range 43–83)
**Tumor grade**	
G1	17 (48.6)
G2	14 (40.0)
G3	4 (11.4)
**Histological type**	Squamous cell carcinoma—35 (100.0)
**HPV status**	
HPV-positive (total)	11 (31.4)
– HPV genotypes 16/18/33	11 (31.4)
HPV-negative	24 (68.6)
Median age by HPV status (years)	
HPV-positive	58.0 (55.0–65.5)
HPV-negative	72.5 (67.8–79.0)
**Surgical procedure**	
Hemivulvectomy ± ipsilateral lymph node dissection ± adjuvant RT	12 (34.3)
Radical total vulvectomy ± adjuvant RT/bilateral LN dissection	6 (17.1)
Radical vulvectomy with separate incisions and bilateral LN dissection	17 (48.6)
Hospital stay (days)	Mean 8.8 ± 2.9 (range 5–15)
Drainage duration (days)	Mean 11.7 ± 6.6 (range 4–29)
**Early complications**	
Neuropathic pain/paresthesia	11 (31.4)
Lymphocysts	6 (17.1)
Postoperative hematoma	3 (8.6)
Wound infection	2 (5.7)
Complete wound dehiscence	1 (2.9)
No early complications	12 (34.3)
**Late complications**	
Lymphedema (mons pubis/lower limbs)	25 (71.4)
Local recurrence	1 (2.9)
No late complications	9 (25.7)

**Legend:** Values are expressed as mean ± standard deviation (SD), median (interquartile range), or number (percentage). RT—radiotherapy; LN—lymph node; HPV—human papillomavirus.

**Table 3:** Postoperative outcomes and complication patterns in patients with vulvar squamous cell carcinoma (n = 35).

Variable	Category	n	Complications n/N (%)	Late Complications n/N (%)	Recurrence n/N (%)	Hospital Stay Median [Mean]	Drainage Duration Median [Mean]
**Overall complications**	Early complications (total)	35	23/35 (65.7)	—	—	—	—
	Late complications (total)	35	—	28/35 (80.0)	—	—	—
Tumor grade	G1–G2	31	19/31 (61.3)	22/31 (71.0)	0 (0.0)	8.0 (7.0–10.0) [8.5]	9.0 (8.0–12.0) [11.1]
	G3	4	4/4 (100.0)	4/4 (100.0)	1 (25.0)	10.5 (7.0–14.2) [10.8]	17.0 (10.8–22.8) [16.5]
Surgical procedure	Hemivulvectomy ± ipsilateral LN dissection/adjuvant RT	12	8/12 (66.7)	9/12 (75.0)	0 (0.0)	8.0 (6.0–9.2) [8.2]	9.5 (7.0–11.2) [11.3]
	Radical total vulvectomy ± adjuvant RT/bilateral LN dissection	6	5/6 (83.3)	5/6 (83.3)	0 (0.0)	10.5 (9.0–13.5) [11.0]	14.0 (12.0–20.5) [16.5]
	Radical vulvectomy with separate incisions and bilateral LN dissection	17	10/17 (58.8)	14/17 (82.4)	1 (5.9)	8.0 (7.0–10.0) [8.4]	9.0 (8.0–12.0) [10.3]
Age group	<65 years	8	5 (62.5)	6 (75.0)	0 (0.0)	8.3	10.6
	≥65 years	27	23 (85.2)	22 (81.5)	1 (3.7)	8.9	12.0
Diabetes	Yes	12	11 (91.7)	10 (83.3)	0 (0.0)	9.5	13.7
	No	23	17 (73.9)	18 (78.3)	1 (4.3)	8.4	10.7
Early vs. late complications	No early complications	12	—	5 (41.7)	0 (0.0)	8.3	10.8
	With early complications	23	—	23 (100.0)	1 (4.3)	9.0	12.2
	No late complications	7	0 (0.0)	—	0 (0.0)	7.9	10.0
	With late complications	28	23 (82.1)	—	1 (3.6)	9.0	12.1
Specific early complications	Neuropathic pain/paresthesia	11	11/35 (31.4)	—	—	—	—
	Lymphocyst	6	6/35 (17.1)	—	—	—	—
	Hematoma	3	3/35 (8.6)	—	—	—	—
	Wound infection	2	2/35 (5.7)	—	—	—	—
	Wound dehiscence	1	1/35 (2.9)	—	—	—	—
Specific late complications	Lymphedema of mons pubis/lower limbs	25	—	25/35 (71.4)	—	—	—
	Local recurrence	1	—	1/35 (2.9)	1/35 (2.9)	—	—

***Legend****:* LN—lymph node; RT—radiotherapy; IQR—interquartile range. Values are presented as median (IQR) [mean] unless otherwise indicated. Early complications occurred in 23/35 patients (65.7%), most frequently neuropathic pain (31.4%) and lymphocyst formation (17.1%). Late morbidity was observed in 28/35 (80.0%), dominated by lower-limb lymphedema (71.4%). Early complications significantly predicted late sequelae (*p* < 0.05).

**Table 4:** Hospitalization, drainage duration, comorbidities, and temporal outcome patterns in patients with vulvar squamous cell carcinoma (n = 35).

Variable	Category	n	Complications n (%)	Recurrence n (%)	Lymphedema n (%)	Mean Hospital Stay (Days ± SD)	Mean Drainage (Days ± SD)	Median (Range)
**Hospitalization**	—	35	—	—	—	8.8 ± 2.9	—	8 (5–15)
**Drainage duration**	—	35	—	—	—	—	11.7 ± 6.6	10 (4–29)
**Cardiovascular disease**	Yes	32	25 (78.1)	—	—	8.9	11.9	—
	No	3	3 (100.0)	—	—	7.7	9.7	—
**Chronic kidney disease**	Yes	1	1 (100.0)	—	—	7.0	12.0	—
	No	34	27 (79.4)	—	—	8.8	11.7	—
**Smoking**	Yes	4	3 (75.0)	—	—	8.8	11.5	—
	No	31	25 (80.6)	—	—	8.8	11.8	—
**Time period**	Pre-2020	18	13 (72.2)	—	—	8.6	10.8	—
	Post-2020	17	15 (88.2)	—	—	9.0	12.6	—
**Drainage duration group**	Short (<10 days)	17	—	1 (5.9)	12 (70.6)	6.9	7.3	—
	Long (≥10 days)	18	—	0 (0.0%)	13 (72.2)	10.6	15.9	

***Legend****:* SD—standard deviation. Values are expressed as mean ± SD and median (range), as appropriate. Cardiovascular disease and diabetes were the most frequent comorbidities. Longer drainage duration (≥10 days) was associated with longer hospitalization but did not significantly affect recurrence or lymphedema rates. After 2020, when separate-incision and ICG-guided techniques were implemented, complication rates slightly increased due to better documentation, but recovery time improved modestly.

**Table 5:** Outcomes by macroscopic aspect (n = 35).

Group	n	G1	G2	G3	Complications n (%)	Mean Hospital Stay (Days)	Mean Drainage (Days)
**Vegetative/ulcerated (mixed)**	32	17	12	3	25 (78.1)	8.6	11.6
**Infiltrative**	3	0	2	1	3 (100.0)	10.7	14.0

**Table 6:** Comparison of HPV-positive and HPV-negative cases (n = 35).

Outcome	HPV-Positive, n (%) (n = 11)	HPV-Negative, n (%) (n = 24)	Test/Effect Size (95% C)
**Age (years)**	Median 58.0 (IQR 55.0–65.5)	Median 72.5 (IQR 67.8–79.0)	Mann–Whitney U = 20.0, ***p*** < 0.001
**Tumor grade G3 (vs. G1–2)**	2/11 (18.2)	2/24 (8.3)	Fisher exact ***p*** = 0.575
**Early complications (any)**	8/11 (72.7)	15/24 (62.5)	Fisher *p* = 0.709; RR = 1.16 (0.72–1.87); RD = +10.2% (−35.4–+47.5)
**Late complications (total)**	8/11 (72.7)	18/24 (75.0)	Fisher *p* = 1.000; RR = 0.97 (0.63–1.49); RD = −2.3% (−44.6–+35.2)
**Hospitalization days**	Median 7.0 (IQR 6.0–10.5)	Median 8.5 (IQR 7.0–10.0)	Mann–Whitney U = 111.5, ***p*** = 0.474; RLM β(HPV+) = +0.41 days, ***p*** = 0.804

***Legend:** RR = risk ratio; RD = risk difference; CI = confidence interval; RLM = robust linear model.*

## Data Availability

The data presented in this study are available on request from the corresponding author. The data are not publicly available due to patient confidentiality.
